# Generalized Maximum Correntropy Cubature Kalman Filter with Variational Bayesian for SINS/GPS Integrated Navigation Systems

**DOI:** 10.3390/s26123961

**Published:** 2026-06-22

**Authors:** Weisheng Ma, Bin Wei, Xi Liu

**Affiliations:** School of Systems Science and Engineering, Sun Yat-sen University, Guangzhou 510275, China; mawsh3@mail2.sysu.edu.cn (W.M.); weib23@mail2.sysu.edu.cn (B.W.)

**Keywords:** variational Bayesian, generalized maximum correntropy criterion, Cubature Kalman filter, SINS/GPS integrated navigation systems

## Abstract

To address the degraded accuracy and poor robustness of Strapdown Inertial Navigation Systems (SINSs)/Global Positioning Systems (GPSs) integrated navigation systems under time-varying non-Gaussian measurement noises, this paper proposes a variational Bayesian generalized maximum correntropy cubature Kalman filter (VBGMCCKF). The proposed method combines variational Bayesian adaptive method with the generalized maximum correntropy criterion, enabling the filter to handle the noises with time-varying statistical characteristics and effectively improving its applicability to different types of non-Gaussian noises. The results under different scenarios demonstrate that VBGMCCKF achieves superior estimation accuracy and robustness in the SINS/GPS integrated navigation systems compared with other existing methods. These results confirm the effectiveness of the proposed method for integrated navigation systems under complex noise environments.

## 1. Introduction

Strapdown Inertial Navigation Systems (SINS) and Global Positioning Systems (GPS) are widely applied across aerospace, marine, and land domains for critical tasks like precise positioning and attitude estimation. Individually, SINS provides high-frequency measurements but suffers from cumulative drift, whereas GPS offers stable long-term referencing but is vulnerable to signal blockages in complex environments. Consequently, integrating SINS with GPS synergistically fuses their complementary strengths and mitigates individual limitations to deliver a continuous, accurate, and robust navigation solution [1,2,3,4]. Such continuous and reliable positioning is a fundamental prerequisite for trajectory-related applications [5].

Data fusion techniques play a pivotal role in the design of integrated navigation systems, with the Kalman filter (KF) being widely adopted for state estimation. With the assumption of Gaussian noise and the minimum mean square error (MMSE) criterion, KF provides an optimal recursive estimator for linear state-space systems [6]. In practice, however, system models are often nonlinear, and the performance of conventional KF may deteriorate significantly. To handle this challenge, several suboptimal filtering strategies are developed within the KF framework, including the Extended Kalman Filter (EKF) [7], Unscented Kalman Filter (UKF) [8], and Cubature Kalman Filter (CKF) [9], which improve estimation accuracy and robustness in nonlinear systems.

Despite their extensive use, the aforementioned filters optimized under the minimum mean square error (MMSE) framework are fundamentally constrained by the assumption of Gaussian noises. In practical applications, the integrated navigation system frequently encounters severe non-Gaussian anomalies, including multipath interference, non-line-of-sight (NLOS) signals, and extreme measurement outliers caused by sudden electromagnetic interference or transient sensor malfunctions [10,11]. To tackle the state estimation challenges in such complex non-Gaussian environments, various robust strategies are progressively introduced. Early interventions primarily rely on Huber’s M-estimation theory, which successfully mitigates the influence of mildly contaminated Gaussian noise [12,13]. Another important class of robust filtering methods are based on the Student’s *t* distribution, which explicitly model heavy-tailed process and/or measurement noises and thus provide a probabilistic way to reduce the impact of outliers [14]. Nevertheless, the performance of Student’s *t*-based filters is often affected by the selection of the degree-of-freedom parameter and the approximation of the posterior distribution. A more versatile paradigm is then developed with the adoption of the maximum correntropy criterion (MCC). By naturally incorporating higher-order statistical moments of the data, the correntropy framework demonstrates strong resilience against non-Gaussian interferences, offering a fundamentally more robust mechanism [15,16].

To address the challenges posed by system nonlinearities and non-Gaussian anomalies, researchers integrate the Gaussian-kernel-based MCC into the Kalman filtering framework. This integration leads to the development of various robust algorithms, notably including the Maximum Correntropy Kalman Filter (MCKF) [17], Maximum Correntropy Extended Kalman Filter (MCEKF) [18,19], Maximum Correntropy Unscented Kalman Filter (MCUKF) [20,21], and Maximum Correntropy Cubature Kalman Filter (MCCKF) [22,23,24]. However, the aforementioned MCC utilizing the standard Gaussian kernel is not necessarily the optimal choice for all types of complex non-Gaussian noises. Consequently, the generalized maximum correntropy criterion (GMCC) is introduced, which adopts the generalized Gaussian density (GGD) as its kernel function [25]. By adjusting the shape parameters of the GGD, GMCC enables the adaptive modeling of a broader spectrum of non-Gaussian noise distributions, offering substantial flexibility for robust state estimation. Leveraging this advanced framework, GMCC-based filtering algorithms are successfully applied to diverse fields, such as complex target tracking [26] and battery state estimation [27,28], effectively mitigating the adverse effects of non-Gaussian disturbances.

However, despite the unparalleled flexibility of the GMCC in handling non-Gaussian noise, the performance of the traditional KF framework heavily relies on the assumption of known and constant noise covariances. In practical integrated navigation systems operating in complex environments, the statistical characteristics of the noises are often time-varying due to the continuous fluctuation in the number of visible satellites and the geometric dilution of precision [29,30,31]. Using fixed nominal covariance matrices under such conditions inevitably leads to degraded filtering accuracy or even divergence. To tackle this challenge, variational Bayesian (VB)-based adaptive filtering methods are introduced to jointly estimate system states and time-varying noise statistics, demonstrating superior performance over traditional filters under unknown or uncertain noise statistics in target tracking and underwater localization problems [32,33].

To effectively handle different types of non-Gaussian measurement noises and adapt to scenarios with time-varying statistical characteristics of noises, a novel variational Bayesian generalized maximum correntropy cubature Kalman filter (VBGMCCKF) is proposed in this paper. The main contributions of this work are summarized as follows: (1) A VBGMCCKF framework is developed by combining the VB-based adaptive method with the GMCC. Specifically, the introduction of GMCC enhances the algorithm’s applicability to various types of non-Gaussian measurement noises, while the incorporation of the VB adaptive method improves the estimation accuracy in scenarios with time-varying statistical characteristics of measurement noises. (2) The effectiveness of the proposed method is verified in SINS/GPS integrated navigation systems under multiple mixed non-Gaussian noise conditions and scenarios involving time-varying statistical characteristics of measurement noises.

The organization of this paper is as follows. In Section 2, we provide a brief review of the generalized maximum correntropy criterion (GMCC) and variational Bayesian (VB) adaptive filtering methods. In Section 3, the proposed VBGMCCKF algorithm is derived. In Section 4, the proposed method is applied to the SINS/GPS integrated navigation systems under different scenarios to demonstrate its superior performance. Finally, Section 5 gives the conclusion.

## 2. Preliminaries

### 2.1. Generalized Maximum Correntropy Criterion

Considering two random variables *X* and *Y*, correntropy is defined by

(1) $$V\left(X,Y\right)=E\left[\kappa \left(X,Y\right)\right]=\;\int \int \;\kappa \left(x,y\right)\;dF_{XY}\left(x,y\right),$$

where E[·] denotes the expectation operator, $F_{XY}\left(x,y\right)$ represents the joint distribution function of *X* and *Y*, and κ(·,·) is a kernel function satisfying Mercer’s condition. In correntropy-based methods, the Gaussian kernel is commonly adopted and is given by

(2) $$\kappa \left(x,y\right)=G_{\sigma }\left(x-y\right)=\frac{1}{\sqrt{2\pi }\sigma }\exp\left(-\frac{e^{2}}{2\sigma^{2}}\right),$$

where e=x−y denotes the error between *x* and *y*, and σ>0 is the kernel bandwidth.

Although the Gaussian-kernel-based correntropy has shown effectiveness in Kalman type filtering for state estimation, Gaussian kernel form may not be universally optimal for handling various types of non-Gaussian noises. To achieve greater adaptability, the Gaussian kernel can be extended to a generalized Gaussian kernel, leading to the generalized correntropy framework. This extension allows the kernel shape to be adjusted according to different noise environments, thereby improving robustness and estimation performance. The generalized-Gaussian-kernel-based correntropy is defined as follows:

(3) $$G_{\alpha ,\beta }\left(e\right)=\frac{\alpha }{2\beta \cdot \Gamma \left(\frac{1}{\alpha }\right)}\exp\left(-{\left|\frac{e}{\beta }\right|}^{\alpha }\right),$$

where α>0 and β>0 represent the shape parameters and bandwidth parameters, respectively. Γ(·) is the gamma function.

### 2.2. Variational Bayesian

#### 2.2.1. System Model and Problem Formulation

In general state estimation problems, the discrete-time dynamic nonlinear system can be described by the following state and measurement equations:

(4) $$x_{k}=f_{k-1}\left(x_{k-1}\right)+w_{k-1},$$

(5) $$z_{k}=h_{k}\left(x_{k}\right)+v_{k},$$

where $x_{k}\in R^{n}$ represents the system state vector at epoch *k*, and $z_{k}\in R^{m}$ is the corresponding measurement vector. The nonlinear functions $f_{k-1}\left(\cdot \right)$ and $h_{k}\left(\cdot \right)$ describe the state transition and measurement processes, respectively. In general, the process noise $w_{k-1}$ and the measurement noise $v_{k}$ are typically assumed to be zero mean with known covariance matrices $Q_{k-1}$ and $R_{k}$, respectively.

However, in complex practical applications, the covariance matrix of measurement noise $R_{k}$ is often unknown or time-varying. In this case, the conventional Kalman type filter experiences performance degradation because it relies on accurate prior knowledge of the noise statistics. Therefore, it is necessary to jointly estimate the system state $x_{k}$ and the unknown measurement noise covariance $R_{k}$, which requires calculating the joint posterior probability density function (PDF) $p(x_{k},R_{k}|z_{1:k})$.

#### 2.2.2. Variational Bayesian Approximation

Since the state $x_{k}$ and the covariance matrix $R_{k}$ are highly coupled in the likelihood function, it is computationally intractable to obtain the exact analytical solution for the joint posterior PDF $p(x_{k},R_{k}|z_{1:k})$. To address this issue, the variational Bayesian (VB) approach is introduced. According to the mean-field theory, the VB method utilizes a free-form approximation to factorize the joint posterior distribution into the product of two mutually independent approximate densities:

(6) $$p\left(x_{k},R_{k}|z_{1:k}\right)\approx q\left(x_{k}\right)q\left(R_{k}\right),$$

where $q(x_{k})$ and $q(R_{k})$ are the unknown approximate PDFs for the state and the covariance matrix, respectively. The optimal forms of these approximate distributions are obtained by minimizing the Kullback–Leibler (KL) divergence between the true joint posterior and its factorized approximation. Based on the calculus of variations, the optimal solutions satisfy the following logarithmic equations:

(7) $$q\left(x_{k}\right)\propto exp\left(\int logp\left(z_{k},x_{k},R_{k}|z_{1:k}\right)q\left(R_{k}\right)dR_{k}\right),$$

(8) $$q\left(R_{k}\right)\propto exp\left(\int logp\left(z_{k},x_{k},R_{k}|z_{1:k}\right)q\left(x_{k}\right)dx_{k}\right).$$

#### 2.2.3. Conjugate Prior and Inverse-Wishart Distribution

In Bayesian statistics, conjugate priors are widely adopted to ensure that the posterior distribution and the prior distribution belong to the same probability distribution family. For a Gaussian distribution with an unknown covariance matrix, the Inverse-Wishart (IW) distribution serves as its conjugate prior. Thus, the approximated joint posterior distribution can be modeled as the product of a Gaussian distribution and an IW distribution:

(9) $$p\left(x_{k},R_{k}|z_{1:k}\right)\approx N\left(x_{k}|{\hat{x}}_{k},P_{k}\right)IW\left(R_{k}|v_{k},V_{k}\right),$$

where the probability densities of the Gaussian distribution and the IW distribution are defined as follows:

(10) $$N\left(x_{k}|{\hat{x}}_{k},P_{k}\right)\propto {\left|P_{k}\right|}^{-1/2}exp\left(-\frac{1}{2}{\left(x_{k}-{\hat{x}}_{k}\right)}^{T}P_{k}^{-1}\left(x_{k}-{\hat{x}}_{k}\right)\right),$$

(11) $$IW\left(R_{k}|v_{k},V_{k}\right)\propto {\left|R_{k}\right|}^{-(\nu_{k}+n+1)/2}\exp\left(-\frac{1}{2}\mathrm{tr}\left(V_{k}R_{k}^{-1}\right)\right),$$

where tr(·) denotes the trace operator of a matrix, $v_{k}$ represents the degrees of freedom parameter, and $V_{k}$ is the inverse scale matrix associated with the IW distribution.

A significant property of the IW distribution is that the mathematical expectation of the inverse covariance matrix has an exact analytical form, which is crucial for subsequent state updating:

(12) $${\langle R_{k}^{-1}\rangle }_{R}=\left(v_{k}-n-1\right)V_{k}^{-1},$$

where ${\langle \cdot \rangle }_{R}$ denotes the expectation with respect to the distribution $q(R_{k})$. By substituting this expectation into the state update equations, the optimal estimates for both the state vector and the noise covariance matrix can be recursively computed.

## 3. Algorithm Derivation and Analysis

Standard Cubature Kalman Filters (CKFs) frequently experience degraded accuracy in non-Gaussian noise environments or time-varying statistical characteristics noise environments. To overcome these obstacles, this paper formulates a novel robust adaptive filter that amalgamates variational Bayesian (VB) learning with the generalized maximum correntropy criterion (GMCC). Rather than employing a static nominal covariance matrix, our method models the covariance matrix of measurement noise $R_{k}$ as a random matrix subject to an Inverse-Wishart (IW) prior. Concurrently, the GMCC is utilized to dynamically weight the measurement residuals, thereby suppressing non-Gaussian noises. Within this unified architecture, the covariance matrix of measurement noise during the update phase is simultaneously governed by the VB-inferred expectation and the GMCC-derived weighting matrix.

### 3.1. Cubature Points-Based Propagation

The proposed methodology adopts cubature points-based propagation framework. The fundamental prediction procedures are outlined below:

#### 3.1.1. Time Update

At the initial epoch k=0, the cubature points are generated as

(13) $$x_{0}^{i}={\hat{x}}_{0}+{\tilde{X}}_{i,0}={\hat{x}}_{0}+S_{0|0}\xi_{i},$$

where $S_{0|0}$ denotes the Cholesky factor of the initial error covariance matrix $P_{0|0}$, and $\xi_{i}$ denotes the *i*-th column of the point set $\xi =\sqrt{n}\left[I_{n}-I_{n}\right]$, with $I_{n}$ being the identity matrix of dimension *n*.

For subsequent epochs k≥1, the cubature points are generated based on the previous posterior estimate ${\hat{x}}_{k-1|k-1}$ and posterior covariance matrix $P_{k-1|k-1}$.

(14) $$x_{k-1}^{i}={\hat{x}}_{k-1|k-1}+S_{k-1|k-1}\xi_{i},\;\mathrm{for}\;i=1,\ldots ,2n,$$

where $S_{k-1|k-1}$ is the Cholesky factor of $P_{k-1|k-1}$.

Following the nonlinear state transition $\chi_{k|k-1}^{i}=f_{k-1}\left(x_{k-1}^{i}\right)$, the predicted state vector ${\hat{x}}_{k|k-1}$ is formulated as

(15) $${\hat{x}}_{k|k-1}=\frac{1}{2n}\sum_{i=1}^{2n}\chi_{k|k-1}^{i}.$$

The predicted error covariance matrix is then computed by

(16) $$P_{k|k-1}=\frac{1}{2n}{\tilde{X}}_{k|k-1}{\tilde{X}}_{k|k-1}^{T}+Q_{k-1},$$

where

(17) $${\tilde{X}}_{k|k-1}=\left[\begin{array}{cccc} \chi_{k|k-1}^{1}-{\hat{x}}_{k|k-1} & \cdots & , & \chi_{k|k-1}^{2n}-{\hat{x}}_{k|k-1} \end{array}\right].$$

#### 3.1.2. Measurement Prediction

The cubature points for the measurement are created via

(18) $$x_{k|k-1}^{i}={\hat{x}}_{k|k-1}+S_{k|k-1}\xi_{i},\;\mathrm{for}\;i=1,\ldots ,2n.$$

By propagating these points through the observation equation $z_{k|k-1}^{i}=h_{k}\left(x_{k|k-1}^{i}\right)$, the predicted measurement ${\hat{z}}_{k|k-1}$ is formulated as

(19) $${\hat{z}}_{k|k-1}=\frac{1}{2n}\sum_{i=1}^{2n}z_{k|k-1}^{i}.$$

The innovation covariance and the cross-covariance matrices are given by

(20) $$P_{zz,k|k-1}=\frac{1}{2n}{\tilde{Z}}_{k|k-1}{\tilde{Z}}_{k|k-1}^{T},$$

(21) $$P_{xz,k|k-1}=\frac{1}{2n}{\tilde{X}}_{k|k-1}{\tilde{Z}}_{k|k-1}^{T},$$

where

(22) $${\tilde{Z}}_{k|k-1}=\left[\begin{array}{ccc} z_{k|k-1}^{1}-{\hat{z}}_{k|k-1} & \cdots & z_{k|k-1}^{2n}-{\hat{z}}_{k|k-1} \end{array}\right].$$

### 3.2. VB-GMCC Fixed-Point Iteration Strategy

Since the state vector, the VB distribution parameters, and the GMCC weights are mutually coupled within the update equations, a fixed-point iteration approach is introduced to approximate the optimal joint posterior $p(x_{k},R_{k}|z_{1:k})$. At epoch *k*, the IW prior parameters are recursively propagated by

(23) $$v_{k}^{\left(0\right)}=\rho \left(v_{k-1}-m-1\right)+m+1,$$

(24) $$V_{k}^{\left(0\right)}=\rho V_{k-1},$$

where ρ∈(0,1] denotes the fading factor and *m* represents the dimension of the measurement. The initial iteration variables are designated as ${\hat{x}}_{k}^{\left(0\right)}={\hat{x}}_{k|k-1}$ and $P_{k}^{\left(0\right)}=P_{k|k-1}$. The internal loop proceeds for j=1,…,N iterations as follows.

Step 1: VB Covariance Estimation. The nominal covariance of measurement noise is derived from the preceding iteration step:

(25) $$R_{k}^{\left(j\right)}=\frac{V_{k}^{\left(j-1\right)}}{v_{k}^{\left(0\right)}-m-1}.$$

Its Cholesky decomposition is expressed as

(26) $$R_{k}^{\left(j\right)}=S_{r,k}^{\left(j\right)}{\left(S_{r,k}^{\left(j\right)}\right)}^{T}.$$

Step 2: GMCC Weight Evaluation. The innovation vector associated with the latest predicted measurement is defined as

(27) $$u_{k}=z_{k}-{\hat{z}}_{k|k-1}.$$

Then left multiplying (27) by ${\left(S_{r,k}^{\left(j\right)}\right)}^{-1}$ yields

(28) $$r_{k}^{\left(j\right)}={\left(S_{r,k}^{\left(j\right)}\right)}^{-1}u_{k}.$$

Let $r_{l,k}^{\left(j\right)}$ signify the l-th scalar component of $r_{k}^{\left(j\right)}$. Employing the generalized Gaussian kernel $G_{\alpha ,\beta }\left(r\right)=\lambda \kappa_{\alpha ,\beta }\left(r\right)=\lambda \exp\left(-{\left|\frac{r}{\beta }\right|}^{\alpha }\right)$, the robust weights are evaluated as

(29) $$\omega_{l,k}^{\left(j\right)}=\kappa_{\alpha ,\beta }\left(r_{l,k}^{\left(j\right)}\right){\left|r_{l,k}^{\left(j\right)}\right|}^{\alpha -2},\;l=1,\cdots ,m.$$

The diagonal GMCC weighting matrix is then constructed:

(30) $$C_{z,k}^{\left(j\right)}=diag\left(\omega_{1,k}^{\left(j\right)},\ldots ,\omega_{m,k}^{\left(j\right)}\right).$$

Step 3: Fused Measurement Update. The GMCC adjustments are integrated with the VB-inferred covariance to formulate a modified covariance matrix of measurement noise:

(31) $${\tilde{R}}_{k}^{\left(j\right)}=S_{r,k}^{\left(j\right)}{\left(C_{z,k}^{\left(j\right)}\right)}^{-1}{\left(S_{r,k}^{\left(j\right)}\right)}^{T}.$$

Incorporating ${\tilde{R}}_{k}^{\left(j\right)}$ into (20), the updated covariance matrix of predicted measurement is computed as

(32) $${\tilde{P}}_{zz,k}^{\left(j\right)}=P_{zz,k|k-1}+{\tilde{R}}_{k}^{\left(j\right)}.$$

For the j-th iteration, the Kalman gain, the refined state estimate, and the corresponding covariance matrix are recursively updated via

(33) $$K_{k}^{\left(j\right)}=P_{xz,k|k-1}{\left({\tilde{P}}_{zz,k}^{\left(j\right)}\right)}^{-1},$$

(34) $${\hat{x}}_{k}^{\left(j\right)}={\hat{x}}_{k|k-1}+K_{k}^{\left(j\right)}u_{k},$$

(35) $$P_{k}^{\left(j\right)}=P_{k|k-1}-K_{k}^{\left(j\right)}{\tilde{P}}_{zz,k}^{\left(j\right)}{\left(K_{k}^{\left(j\right)}\right)}^{T}.$$

Step 4: Inverse Scale Matrix Refinement. The VB scale matrix is updated utilizing the newly acquired state estimation:

(36) $$x_{k}^{i,(j)}={\hat{x}}_{k}^{\left(j\right)}+S_{k}^{\left(j\right)}\xi_{i},\;i=1,\ldots ,2n,$$

where $S_{k}^{\left(j\right)}$ is the Cholesky factor of $P_{k}^{\left(j\right)}$.

(37) $$A_{k}^{\left(j\right)}=\frac{1}{2n}\sum_{i=1}^{2n}\left(z_{k}-h_{k}\left(x_{k}^{i,(j)}\right)\right){\left(z_{k}-h_{k}\left(x_{k}^{i,(j)}\right)\right)}^{T},$$

(38) $$V_{k}^{\left(j\right)}=V_{k}^{\left(0\right)}+A_{k}^{\left(j\right)}.$$

The fixed-point loop terminates either after preset *N* cycles or once the norm $\frac{∥{\hat{x}}_{k}^{\left(j\right)}-{\hat{x}}_{k}^{\left(j-1\right)}∥}{∥{\hat{x}}_{k}^{\left(j-1\right)}∥}$ falls below a specified small value. The finalized outputs for the current epoch *k* are established as ${\hat{x}}_{k|k}={\hat{x}}_{k}^{\left(N\right)}$, $P_{k|k}=P_{k}^{\left(N\right)}$, $v_{k}=v_{k}^{\left(0\right)}+1$, and $V_{k}=V_{k}^{\left(N\right)}$, preparing for the subsequent recursion. The VBGMCCKF algorithm is summarized in Algorithm 1.
**Algorithm 1** VBGMCCKF.    **Input:** $z_{k}$, f(·), h(·), $Q_{k-1}$, ρ, GMCC parameters (α,β), Iteration *N*    **Output:** ${\hat{x}}_{k|k}$, $P_{k|k}$, $v_{k}$, $V_{k}$    **Time update**  1:Generate cubature points $x_{k-1}^{i}$ from ${\hat{x}}_{k-1|k-1}$ and $P_{k-1|k-1}$ using Equation (14).  2:Propagate points $\chi_{k|k-1}^{i}=f_{k-1}\left(x_{k-1}^{i}\right)$ and evaluate predicted state ${\hat{x}}_{k|k-1}$ using Equation (15).  3:Compute predicted error covariance $P_{k|k-1}$ using Equation (16).    **Measurement update**  4:Generate cubature points $x_{k|k-1}^{i}$ using Equation (18).  5:Calculate $z_{k|k-1}^{i}=h_{k}\left(x_{k|k-1}^{i}\right)$ and compute ${\hat{z}}_{k|k-1}$, $P_{zz,k|k-1}$ and $P_{xz,k|k-1}$ using Equations (19)–(21).  6:Initialize VB parameters: $v_{k}^{\left(0\right)}=\rho \left(v_{k-1}-m-1\right)+m+1$ and $V_{k}^{\left(0\right)}=\rho V_{k-1}$.  7:Set initial iteration values: ${\hat{x}}_{k}^{\left(0\right)}={\hat{x}}_{k|k-1}$ and $P_{k}^{\left(0\right)}=P_{k|k-1}$.  8:**for** j=1 to *N* **do**  9:     Update measurement noise covariance $R_{k}^{\left(j\right)}$ using Equation (25).10:     Calculate $r_{k}^{\left(j\right)}$ and GMCC weight matrix $C_{z,k}^{\left(j\right)}$ using Equations (28)–(30).11:     Correct ${\tilde{R}}_{k}^{\left(j\right)}=S_{r,k}^{\left(j\right)}{\left(C_{z,k}^{\left(j\right)}\right)}^{-1}{\left(S_{r,k}^{\left(j\right)}\right)}^{T}$ using Equation (31).12:     Update covariance matrix ${\tilde{P}}_{zz,k}^{\left(j\right)}$ using Equation (32).13:     Update $K_{k}^{\left(j\right)}$, ${\hat{x}}_{k}^{\left(j\right)}$ and $P_{k}^{\left(j\right)}$ using Equations (33)–(35).14:     Refine VB scale matrix $V_{k}^{\left(j\right)}=V_{k}^{\left(0\right)}+A_{k}^{\left(j\right)}$ using Equations (36)–(38).15:     **if** $\frac{∥{\hat{x}}_{k}^{\left(j\right)}-{\hat{x}}_{k}^{\left(j-1\right)}∥}{∥{\hat{x}}_{k}^{\left(j-1\right)}∥}<\epsilon$ **then**16:      **break**17:Finalize: ${\hat{x}}_{k|k}={\hat{x}}_{k}^{\left(N\right)},P_{k|k}=P_{k}^{\left(N\right)},v_{k}=v_{k}^{\left(0\right)}+1,V_{k}=V_{k}^{\left(N\right)}$.


## 4. Algorithm Verification

To evaluate the performance of the proposed VBGMCCKF, the following experiments are conducted in SINS/GPS integrated navigation systems based on the PSINS toolbox.

### 4.1. System Model and Experimental Conditions

The PSINS toolbox (Version 20250418; developed by Gongmin Yan, Xi’an, China) is utilized in this paper to simulate the SINS/GPS integrated navigation experiments of a unmanned aerial vehicle (UAV). The coordinate frames used in the experiments are defined as follows. The body frame (*b*-frame) is attached to the UAV, with its origin placed at the center of mass. A right-handed inertial frame is used as the reference frame. The navigation frame (*n*-frame) is defined as the local geographic East-North-Up (ENU) coordinate system. The SINS operates at 100 Hz, while GPS observations are available at 5 Hz. The inertial measurement unit (IMU) error parameters are set to emulate the performance of a tactical-grade IMU. The gyroscope constant bias is set to 0.03 °/h, with an angular random walk of 0.001 °/h. The accelerometer constant bias is set to 100 μg, with a velocity random walk of 5 μg/Hz.

**Figure 1:**
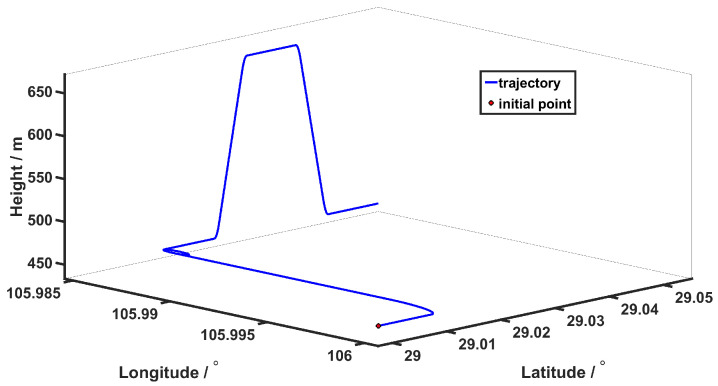
The true trajectory of UAV.

The true trajectory of the UAV used in the experiment is shown in Figure 1.

The 15-dimensional state vector used in the navigation systems is written as

(39) $$x={\left[\phi^{T},{\left(\delta v^{n}\right)}^{T},{\left(\delta p\right)}^{T},\epsilon^{T},\nabla^{T}\right]}^{T},$$

where ϕ is the attitude error, $\delta v^{n}$ is the velocity error, δp is the position error, and ϵ and ∇ are the gyroscope and accelerometer biases, respectively. The state propagation model follows the nonlinear SINS error equations.

The measurement model is constructed based on the position difference between SINS and GPS outputs; then, we can have the following measurement model:

(40) $$\left[\begin{array}{c} L_{SINS}-L_{GPS}\\ \lambda_{SINS}-\lambda_{GPS}\\ H_{SINS}-H_{GPS} \end{array}\right]=\left[\begin{array}{c} \delta L\\ \delta \lambda \\ \delta H \end{array}\right]+\left[\begin{array}{c} r_{1}\\ r_{2}\\ r_{3} \end{array}\right],$$

where $r_{1}$, $r_{2}$ and $r_{3}$ are white measurement noise, and *L*, λ and *H* denote the latitude, longitude and height.

All experiments are conducted with 100 independent Monte Carlo runs to ensure statistical reliability. The estimation performance of different algorithms is quantified by the average mean square error (AMSE), which is defined as follows:

(41) $$\mathrm{MSE}\left(k\right)=\frac{1}{M}\sum_{m=1}^{M}{\left(x\left(k\right)-\hat{x}\left(k|k\right)\right)}^{2},k=1,\ldots ,K,$$

(42) $$\mathrm{AMSE}=\frac{1}{K}\sum_{k=1}^{K}\mathrm{MSE}\left(k\right),$$

where *M* represents the total number of Monte Carlo runs.

### 4.2. Results and Discussion

In practical flight missions, the GNSS receiver onboard the UAV is susceptible to multipath interference, non-line-of-sight (NLOS) signals attenuation caused by blockage from buildings or foliage, and extreme measurement outliers induced by sudden electromagnetic interference from onboard electronics or transient sensor malfunctions. Consequently, the measurement noise often deviates from a standard Gaussian distribution. We consider a composite noise model which combines two independent noise processes, taking the form of

(43) $$v\left(k\right)=\left(\begin{array}{c} 1-\theta (k) \end{array}\right)B\left(k\right)+\theta \left(k\right)D\left(k\right),$$

where θ(k) is a binary Bernoulli random variable taking values in 0 or 1. Specifically, it satisfies p(θ(k)=1)=c,p(θ(k)=0)=1−c, with the parameter *c* controlling the mixing ratio between noise processes B(k) and D(k). In practice, B(k) denotes the nominal noise disturbance with zero mean and small variance, whereas D(k) corresponds to occasional outliers with zero mean and larger variance. Unless otherwise specified, *c* is set to 0.15 in the following experiments. In this paper, B(k) is chosen as Gaussian noise, while D(k) represents the higher-variance noise component, such as Gaussian, Laplace, Uniform, or Binary noise.

In this section, we assume the measurement noises obey the following model:

(44) $$v\left(k\right)=\left(1-c\right)N\left(0,R_{1}\right)+cD\left(0,R_{2}\right),$$

where the covariance matrices $R_{1}$ and $R_{2}$ are set proportional to the nominal covariance matrix $R_{0}$. The nominal covariance matrix is

(45) $$R_{0}=diag\left(\sigma_{lat}^{2},\sigma_{lon}^{2},\sigma_{hgt}^{2}\right),$$

where $\sigma_{lat}=\sigma_{lon}=1.0$ m and $\sigma_{hgt}=3.0$ m.

Meanwhile, in real integrated navigation scenarios, measurement noise is generally nonstationary, since satellite visibility and observation geometry vary continuously throughout the mission. To further emulate realistic signal degradation, the noise amplitude is magnified by a factor of five during the intervals [200, 220] s and [500, 550] s, thereby introducing localized time-varying measurement disturbances. In this way, the experiments simultaneously evaluate robustness against non-Gaussian noise and adaptability to abrupt changes in the statistical characteristic of measurement noise. Five filters, including CKF, variational Bayesian-based CKF (VBCKF), maximum correntropy CKF (MCCKF), generalized maximum correntropy CKF (GMCCKF), and the proposed VBGMCCKF are used for comparison. In addition, four mixed-noise scenarios are investigated, including Gaussian–Gaussian mixture noise, Gaussian–Laplace mixture noise, Gaussian–Uniform mixture noise, and Gaussian–Binary mixture noise.

#### 4.2.1. Case A: Gaussian–Gaussian Mixture Noise

In this case, we consider the following measurement noise:

(46) $$0.9N\left(0,10R_{0}\right)+0.1N\left(0,100R_{0}\right)$$

**Figure 2:**
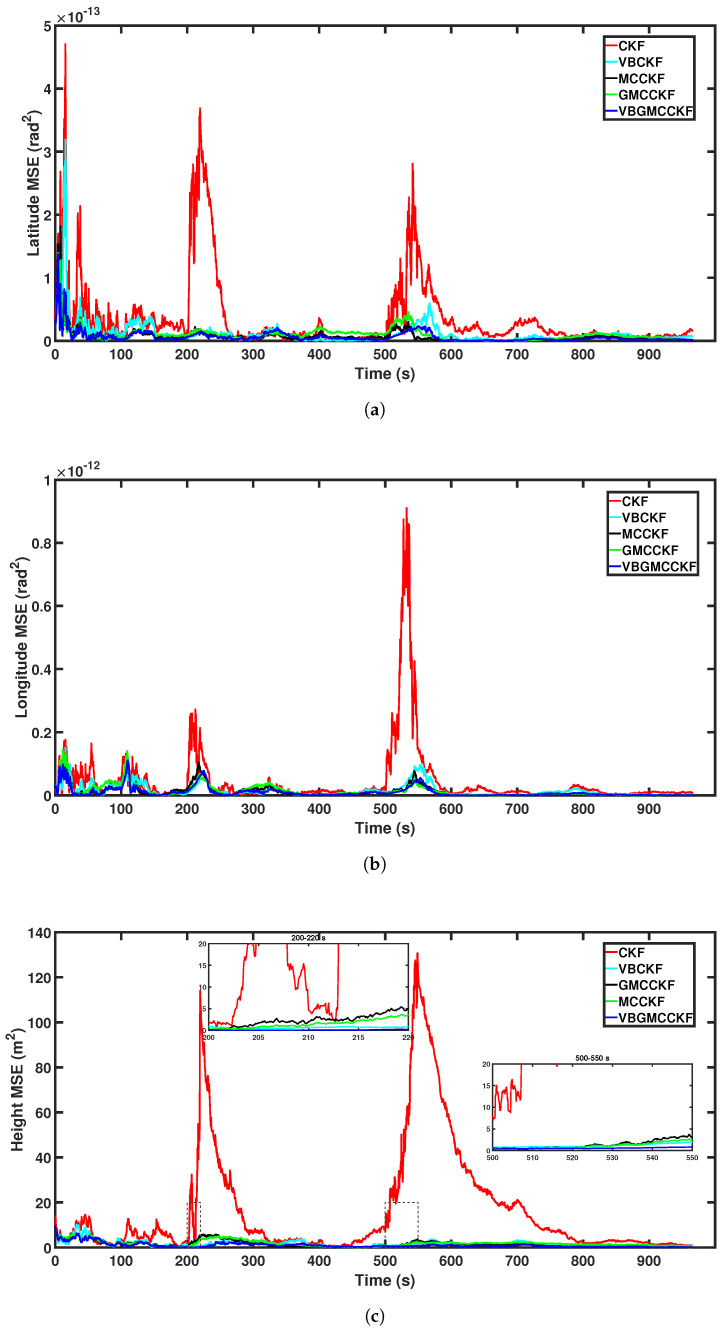
MSEs under Gaussian–Gaussian mixture measurement noise: (**a**) Latitude. (**b**) Longitude. (**c**) Height.

Figure 2 shows the MSEs with different methods under Gaussian–Gaussian mixture measurement noise, and Table 1 lists the corresponding AMSEs. The results indicate that CKF plays the worst performance, especially suffering from significant error peaks during the noise amplification intervals and exhibiting slow recovery afterward. VBCKF improves performance by adapting the noise covariance. MCCKF and GMCCKF reduce error through correntropy-based weighting. Under time-varying Gaussian–Gaussian mixture noise, the GMCCKF does not exhibit superior performance to the MCCKF, when the kernel shape parameter is set to 1.7. In this case, the VBGMCCKF achieves its best performance when the kernel shape parameter is set to 2 and the kernel bandwidth is set to 2, and outperforms other methods.

Table 2 further reports the average iteration numbers and average single-step computational times of different methods. Although the VB-based filters require iterative updates, their average iteration numbers remain around 2.7, and the average single-step times are still far below the 10 ms sampling interval corresponding to a 100 Hz inertial update rate. Therefore, the proposed VBGMCCKF requires slightly more computation time than other algorithms but still satisfies the real-time requirement in this experiment.

#### 4.2.2. Case B: Gaussian–Laplace Mixture Noise

In this case, we consider the following measurement noise:

(47) $$0.85N\left(0,10R_{0}\right)+0.15L\left(0,40R_{0}\right)$$

**Figure 3:**
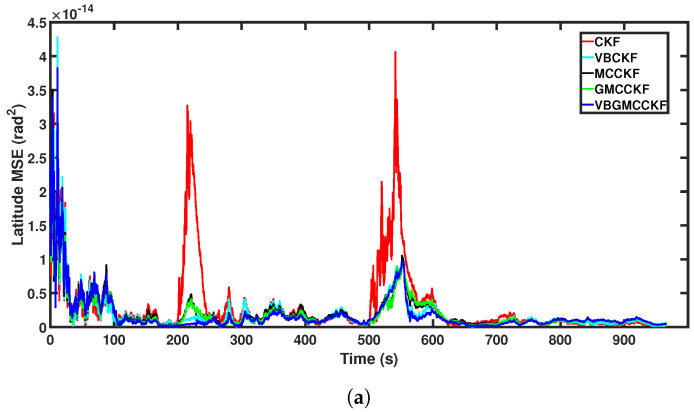

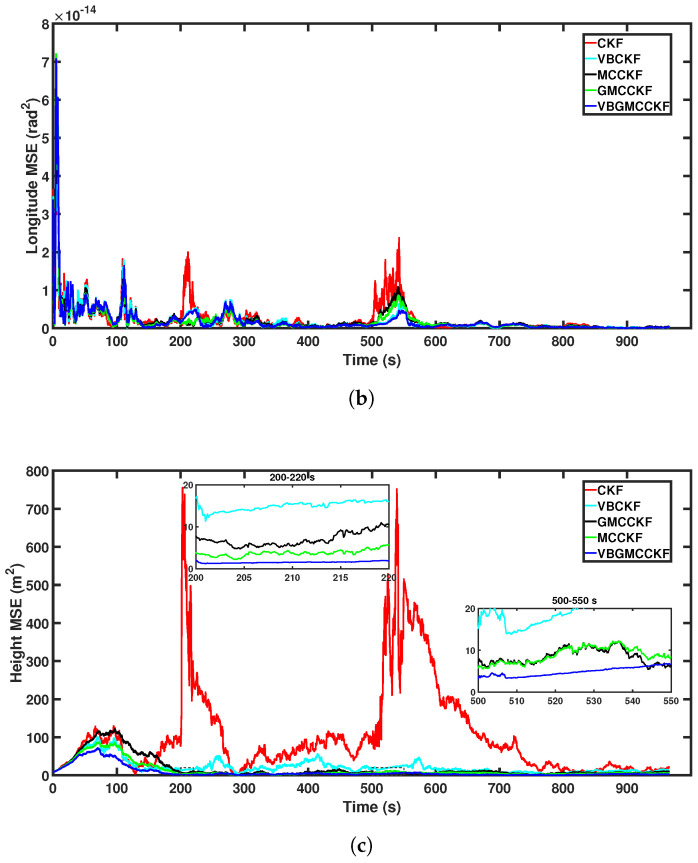
MSEs under Gaussian–Laplace mixture measurement noise: (**a**) Latitude. (**b**) Longitude. (**c**) Height.

Figure 3 shows the MSEs with different methods under Gaussian–Laplace mixture measurement noise, and Table 3 lists the corresponding AMSEs. A trend comparable to that observed in Case A can still be found. In particular, the VBGMCCKF achieves its best performance when the kernel shape parameter is set to 1.8 and the kernel bandwidth is set to 3, and outperforms other methods under Gaussian–Laplace mixture measurement noise. In addition, the benefit of VBGMCCKF is more visible in the height component.

#### 4.2.3. Case C: Gaussian–Uniform Mixture Noise

In this case, we consider the following measurement noise:

(48) $$0.85N\left(0,10R_{0}\right)+0.15U\left(-20R_{0},20R_{0}\right)$$

**Figure 4:**
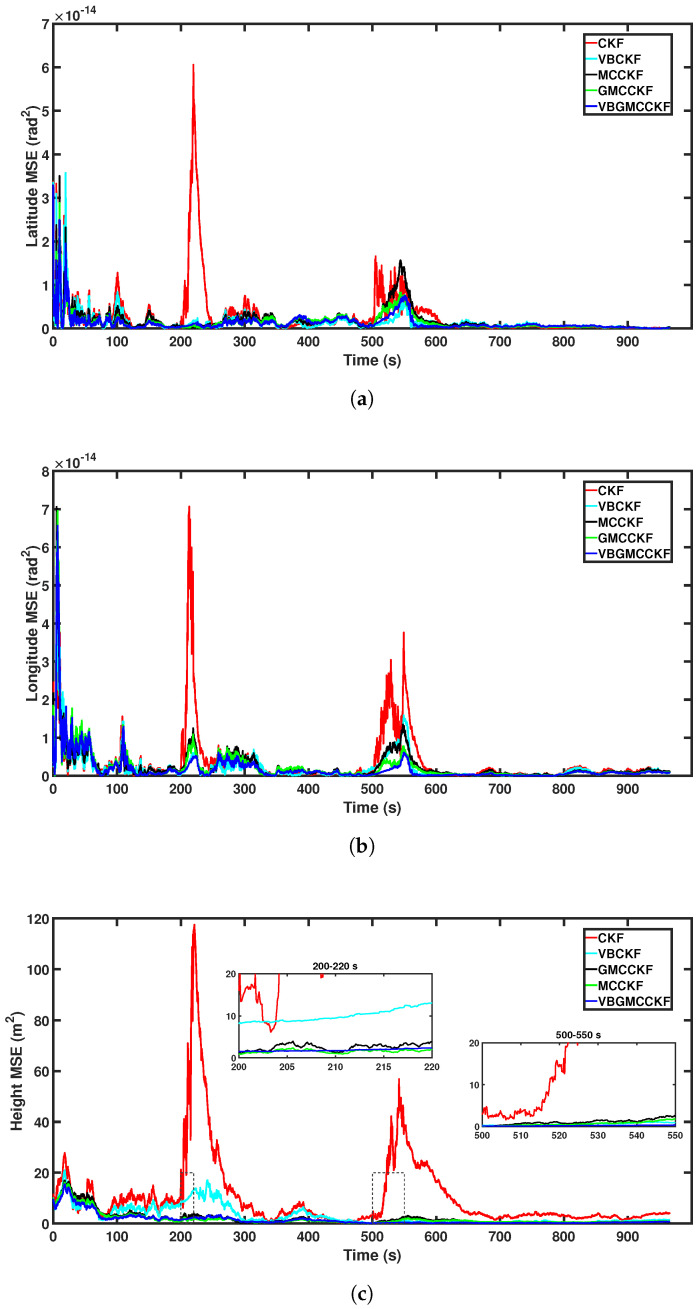
MSEs under Gaussian–Uniform mixture measurement noise: (**a**) Latitude. (**b**) Longitude. (**c**) Height.

Figure 4 shows the MSEs with different methods under Gaussian–Uniform mixture measurement noise, and Table 4 lists the corresponding AMSEs. Similar behavior can also be observed in this case. It should be noted that VBGMCCKF attains its best performance when the kernel shape parameter is set to 1.7 and the kernel bandwidth is set to 4, and remains the most effective method among those considered under Gaussian–Uniform mixture measurement noise.

#### 4.2.4. Case D: Gaussian–Binary Mixture Noise

In this case, we consider the following measurement noise:

(49) $$0.85N\left(0,10R_{0}\right)+0.15Binary\left(-20R_{0},20R_{0}\right)$$

**Figure 5:**
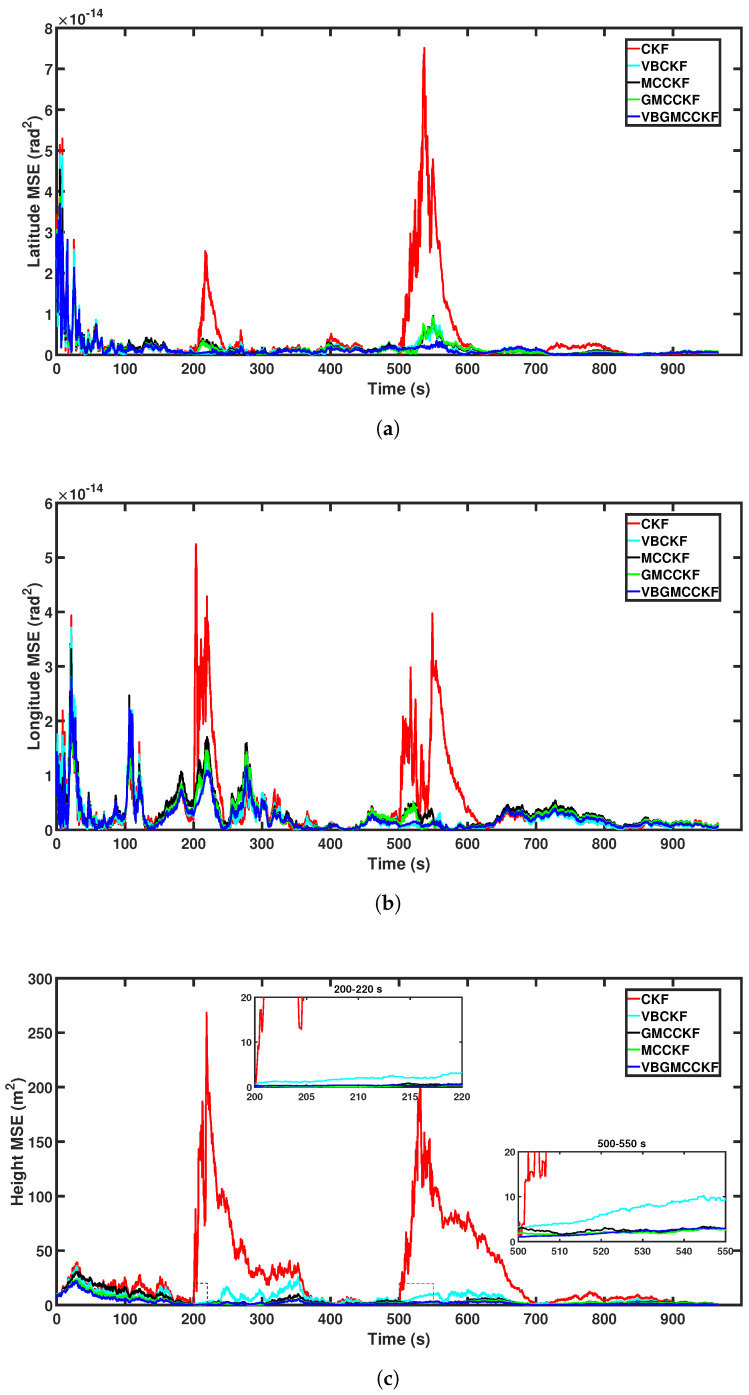
MSEs under Gaussian–Binary mixture measurement noise: (**a**) Latitude. (**b**) Longitude. (**c**) Height.

Figure 5 shows the MSEs with different methods under Gaussian–Binary mixture measurement noise, and Table 5 lists the corresponding AMSEs. The results are similar to the previous case. It is noted that the VBGMCCKF achieves its best performance when the kernel shape parameter is set to 1.8 and the kernel bandwidth is set to 4, and outperforms other methods under Gaussian–Binary mixture measurement noise.

These results indicate that using only covariance-based adaptation or only robust weighting is insufficient to handle complex measurement noises characterized by both non-Gaussianity and time-varying properties. However, combining VB-based adaptive noise estimation with GMCC-based robust updates can provide superior performance in these cases. Moreover, with appropriate selection of the kernel parameters, GMCC offers improved adaptability to different types of non-Gaussian noises. For deployment in unknown noise environments, based on the kernel parameters in this paper as reference values, offline residual or fitting distribution analysis of repeated experimental data can be used for setting or tuning the parameters.

## 5. Conclusions

This paper proposes a variational Bayesian generalized maximum correntropy cubature Kalman filter (VBGMCCKF) for integrated navigation systems in time-varying non-Gaussian measurement noises. This method combines the variational Bayesian approach with generalized maximum correntropy criterion, enabling better handling of time-varying statistical characteristics noise and non-Gaussian disturbances. Experimental results under various scenarios show that the VBGMCCKF has higher estimation accuracy and stronger robustness compared to other existing methods. It should also be noted that the proposed VBGMCCKF requires slightly more computation time than the compared algorithms but still within an acceptable range. In future work, we will further validate its performance using real-world UAV datasets under three-dimensional motion, high-frequency vibration, and aggressive maneuver conditions, and investigate adaptive kernel-parameter selection methods.

## Figures and Tables

**Table 1:** AMSEs under Gaussian–Gaussian mixture measurement noise.

Method	Latitude (m^2^)	Longitude (m^2^)	Height (m^2^)
CKF	1.436	1.337	16.377
VBCKF	0.453	0.453	1.670
MCCKF	0.278	0.361	1.474
GMCCKF (α=1.7, β=2)	0.390	0.403	1.795
VBGMCCKF (α=2.2, β=2)	0.260	0.327	1.072
VBGMCCKF (α=1.8, β=2)	0.252	0.319	1.137
VBGMCCKF (α=2, β=2)	0.246	0.301	0.985

**Table 2:** Average iteration numbers and average computational times.

Method	Average Iteration Number	Average Computational Time (ms)
CKF	-	0.0507
VBCKF	2.6522	0.0830
MCCKF	-	0.0515
GMCCKF (α=1.7, β=2)	-	0.0524
VBGMCCKF (α=2.2, β=2)	2.6934	0.0897
VBGMCCKF (α=1.8, β=2)	2.7022	0.0894
VBGMCCKF (α=2, β=2)	2.6750	0.0872

**Table 3:** AMSEs under Gaussian–Laplace mixture measurement noise.

Method	Latitude (m^2^)	Longitude (m^2^)	Height (m^2^)
CKF	0.124	0.080	103.668
VBCKF	0.074	0.060	21.953
MCCKF	0.080	0.067	18.385
GMCCKF (α=1.8, β=3)	0.076	0.061	12.989
VBGMCCKF (α=1.8, β=3)	0.069	0.056	8.443

**Table 4:** AMSEs under Gaussian–Uniform mixture measurement noise.

Method	Latitude (m^2^)	Longitude (m^2^)	Height (m^2^)
CKF	0.113	0.114	10.410
VBCKF	0.057	0.066	3.106
MCCKF	0.071	0.076	1.926
GMCCKF (α=1.7, β=4)	0.056	0.066	1.715
VBGMCCKF (α=1.7, β=4)	0.050	0.055	1.452

**Table 5:** AMSEs under Gaussian–Binary mixture measurement noise.

Method	Latitude (m^2^)	Longitude (m^2^)	Height (m^2^)
CKF	0.162	0.137	28.159
VBCKF	0.063	0.137	6.218
MCCKF	0.069	0.094	4.630
GMCCKF (α=1.8, β=4)	0.062	0.081	3.211
VBGMCCKF (α=1.8, β=4)	0.054	0.072	2.504

## Data Availability

The original contributions presented in this study are included in the article. Further inquiries can be directed to the corresponding author.
